# Conserved Synthetic Peptides from the Hemagglutinin of Influenza Viruses Induce Broad Humoral and T-Cell Responses in a Pig Model

**DOI:** 10.1371/journal.pone.0040524

**Published:** 2012-07-16

**Authors:** Júlia Vergara-Alert, Jordi M. Argilaguet, Núria Busquets, Maria Ballester, Gerard E. Martín-Valls, Raquel Rivas, Sergio López-Soria, David Solanes, Natàlia Majó, Joaquim Segalés, Veljko Veljkovic, Fernando Rodríguez, Ayub Darji

**Affiliations:** 1 Centre de Recerca en Sanitat Animal, Universitat Autònoma de Barcelona–Institut de Recerca i Tecnologia Agroalimentària, Campus de la Universitat Autònoma de Barcelona, Bellaterra (Cerdanyola del Vallès), Spain; 2 Departament de Sanitat i Anatomia Animals, Universitat Autònoma de Barcelona, Bellaterra, Barcelona, Spain; 3 Center for Multidisciplinary Research, Institute of Nuclear Sciences VINCA, University of Belgrade, Belgrade, Serbia; 4 Institut de Recerca i Tecnologia Agroalimentàries, Barcelona, Spain; University of Georgia, United States of America

## Abstract

Outbreaks involving either H5N1 or H1N1 influenza viruses (IV) have recently become an increasing threat to cause potential pandemics. Pigs have an important role in this aspect. As reflected in the 2009 human H1N1 pandemia, they may act as a vehicle for mixing and generating new assortments of viruses potentially pathogenic to animals and humans. Lack of universal vaccines against the highly variable influenza virus forces scientists to continuously design vaccines *à la carte*, which is an expensive and risky practice overall when dealing with virulent strains. Therefore, we focused our efforts on developing a broadly protective influenza vaccine based on the Informational Spectrum Method (ISM). This theoretical prediction allows the selection of highly conserved peptide sequences from within the hemagglutinin subunit 1 protein (HA1) from either H5 or H1 viruses which are located in the flanking region of the HA binding site and with the potential to elicit broader immune responses than conventional vaccines. Confirming the theoretical predictions, immunization of conventional farm pigs with the synthetic peptides induced humoral responses in every single pig. The fact that the induced antibodies were able to recognize *in vitro* heterologous influenza viruses such as the pandemic H1N1 virus (pH1N1), two swine influenza field isolates (SwH1N1 and SwH3N2) and a H5N1 highly pathogenic avian virus, confirm the broad recognition of the antibodies induced. Unexpectedly, all pigs also showed T-cell responses that not only recognized the specific peptides, but also the pH1N1 virus. Finally, a partial effect on the kinetics of virus clearance was observed after the intranasal infection with the pH1N1 virus, setting forth the groundwork for the design of peptide-based vaccines against influenza viruses. Further insights into the understanding of the mechanisms involved in the protection afforded will be necessary to optimize future vaccine formulations.

## Introduction

In the last decades, several cases of human infection with the highly pathogenic avian influenza virus (HPAIV) H5N1 have been reported by the World Health Organization http://www.who.int/influenza/human_animal_interface/avian_influenza/en/). It is a common assumption that the pig may act as mixing vessel to generate new reassortant influenza viruses due to the presence of receptors for both avian and mammalian influenza viruses in the epithelial cells of their respiratory tract [1]. A recent example of the latter caused the first pandemia of the 21st century, starting in 2009 as a consequence of the global spread of a swine-origin influenza virus A H1N1 (pH1N1). This was a virus that contained genes from avian, pig and human origin [2]. Although the virus was not as pathogenic to humans as expected, severe disease cases associated with pH1N1 have been more recently reported in England (http://www.who.int/influenza/surveillance_monitoring/updates/2010_12_30_GIP_surveillance/en/). The future evolution of this or any emergent influenza virus (IV) is uncertain. This is a distressing matter particularly because available vaccines and therapies are strictly restricted to phylogenetically closely related circulating viruses. Therefore, finding universal and effective vaccines and therapeutic measures to fight against future IV is a must for public health.

IV hemagglutinin (HA) is a viral surface polypeptide that mediates both, the binding of IV to the host cell surface and the fusion of viral and endosomal membranes [3]. HA is formed by subunit 1 (HA1) and subunit 2 (HA2) and both the N- and C- terminal parts of HA1 together with HA2 comprise the stalk of the molecule [4]. Vaccines designed to elicit antibodies against the stalk of HA are reported to confer protection against IV infection in mice [5]. HA1, although highly variable, encodes specific and highly conserved domains which may be involved in determining the recognition and targeting (RTD) of influenza viruses to their receptor as revealed by the Informational Spectrum Method (ISM) [6]. This includes the VIN1 domain, located within the site E in the N-terminus of HA1 [7]. In contrast with the high variability suffered by the globular part of the HA1 molecule, which is directly responsible for the receptor tropism, the site E remains relatively highly conserved [8]. Thus, representing potential targets to develop broad array of protective therapies and vaccines against IV infection.

Due to the already mentioned recent **cases** related to H5N1 and H1N1 IV subtypes, and because their potential to cause future outbreaks among the population, we focused our efforts on designing a vaccine capable of confering protection against both viral subtypes. As previously reported, RTD of HA1 from different H1N1 strains and HA1 from the recently emerged in Egypt H5N1 IV encode the same information. However, HA1 from H3N2 and all other H5N1 viruses encode different RT information [6], [7]. Thus, aiming to increase the vaccine coverage, one HA1-peptide from the VIN1 domain of H1N1 and three HA1-peptides from two different H5N1 IV strains were designed and selected based on ISM.

In order to test the immunogenicity of our experimental vaccine, we decided to immunize conventional pigs with the combination of the synthesized peptides. Pigs allow the evaluation of the protective efficacy of experimental vaccines against several viral strains, including the recently pandemic H1N1 virus, pH1N1 [11]. Confirming the rationale behind their use as a pre-clinical animal model, immunization of conventional pigs with the VIN1-peptide cocktail allow us to demonstrate the induction of peptide-specific antibody and T-cell responses in every single animal, independently of their swine leukocyte antigen (SLA)-haplotype. Specific B and T-cell responses were induced against each one of the H1 and H5-peptides used, confirming their immunogenicity *in vivo.* Interestingly, the elicited antibodies also recognized several heterologous viruses *in vitro*, including the pH1N1, two swine influenza field isolates (SwH1N1 and SwH3N2) and a H5N1 highly pathogenic avian virus. This, together with the fact that the specific T-cell responses induced were also able to recognize the inactivated pH1N1, encouraged us to challenge all pigs with the pH1N1 influenza virus. Albeit preliminary, our results demonstrate that VIN1-vaccination was able to confer a partial protection against intranasal challenge with pH1N1, as demonstrated with the partial and total viral clearance from the lung lavages in two out of four immunized pigs. We believe that our results could contribute to the obtainment of a broader array of protective vaccines against future influenza outbreaks or even pandemics.

## Results

### VIN1 as a Synthetic Peptide-vaccine

The highly conserved VIN1 domain, located within the E site in the N-terminus of the HA1 molecule, plays an important role in the recognition and targeting (RT) between virus and receptor, therefore representing an ideal target for an antibody-mediated therapy against influenza infection [7]. Informational spectral analysis revealed that the RT domains of HA1 from H1N1/1918, pH1N1/2009, seasonal H1N1 and H5N1 emerged in Egypt encode the same information despite differences in their primary structures. Thus, based on ISM and using information available on the properties of HA and its receptors, a single 34-mer peptide (NF-34) from the H1N1 subtype was selected from within the VIN1 region. NF-34 corresponds to positions 87–120 from the A/South Carolina/1/18 (H1N1) virus (Table 1). Additionally, a peptide (ES-34) from the VIN1 domain from the A/Egypt/0636-NAMRU3/2007 (E; H5N1) was also selected and included in the vaccine. In previous studies, we also showed that HA1 from H3N2 and all other H5N1 encode different RT information [6], [7]. Aiming to increase the vaccine coverage, two additional peptides (LE-35.1 and LE-35.2) were selected from the A/Hong Kong/213/03 (HK; H5N1) IV and both peptides were added to NF-34 and ES-34. LE-35.1 and LE-35.2 differ only in positions 43 and 48 (Table 1), representing “hot spots” of variability within this H5N1 sequence.

**Table 1:** Amino acid sequences from the peptides used for immunization compared to the homologue sequence of the HA receptor recognition domain of the challenging strain (pH1N1) and the HA purified proteins used for the serologic tests.

Strain	Short name	Residues	Sequence
**Challenge**			
A/Catalonia/63/2009 (H1N1)	pH1N1	59–92	**S**S**D**NGTCYPGDFIDYEELREQLSSVSSFE**R**FEIF
**Immunization**			
A/South Carolina/1/18 (H1N1)	NF-34	87–120	**N**S**E**NGTCYPGDFIDYEELREQLSSVSSFE**K**FEIF
A/Egypt/0636-NAMRU3/2007 (H5N1)	ES-34	99–132	EELKHLLSRINHFEKIQIIPK**N**SWS**D**HEA**S**GVSS
A/Hong Kong/213/03 (H5N1)	LE-35.1	41–75	LC**D**LDGV**H**PLILRDCSVAGWLLGNPMCDEFINVPE
A/Hong Kong/213/03 (H5N1)	LE-35.2	41–75	LC**N**LDGV**K**PLILRDCSVAGWLLGNPMCDEFINVPE
**HA purified proteins**			
A/VietNam/1203/04 (H5)	VN04	115–149 57–91	EELKHLLSRINHFEKIQIIPKSSWSSHEASLGVSS LCDLDGVKPLILRDCSVAGWLLGNPMCDEFINVPE
A/New Caledonia/20/99 (H1)	NCD99	101–134	NPENGTCYPGYFADYEELREQLSSVSSFERFEIF

In bold type, the amionacids differences between sequences are represented. Differences between the pH1N1 virus and the H1-peptide (NF-34) in homologous positions within the HA receptor recognition domain are marked. Aminoacid differences in the two H5-HK derived peptides (LE-35.1/2) are also represented.

Amino acid sequences from pH1N1 virus and VIN1-peptides are given in Table 1. The identity between pH1N1 virus and NF-34 (H1-peptide) is 92%. The similarity between all the H5-peptides and the pH1N1 virus is less than 75% with even lower identities, being less than 40% when comparing pH1N1 and ES-34. There was no homology between the HK-derived peptides (LE-35.1 and LE-35.2) and the E-derived peptide (ES-34). The two amino acid differences between LE-35.1 and LE-35.2 represent a 95% of identity between them.

### Immunogenicity of VIN1 Peptides in a Pig Model

**Figure 1:**
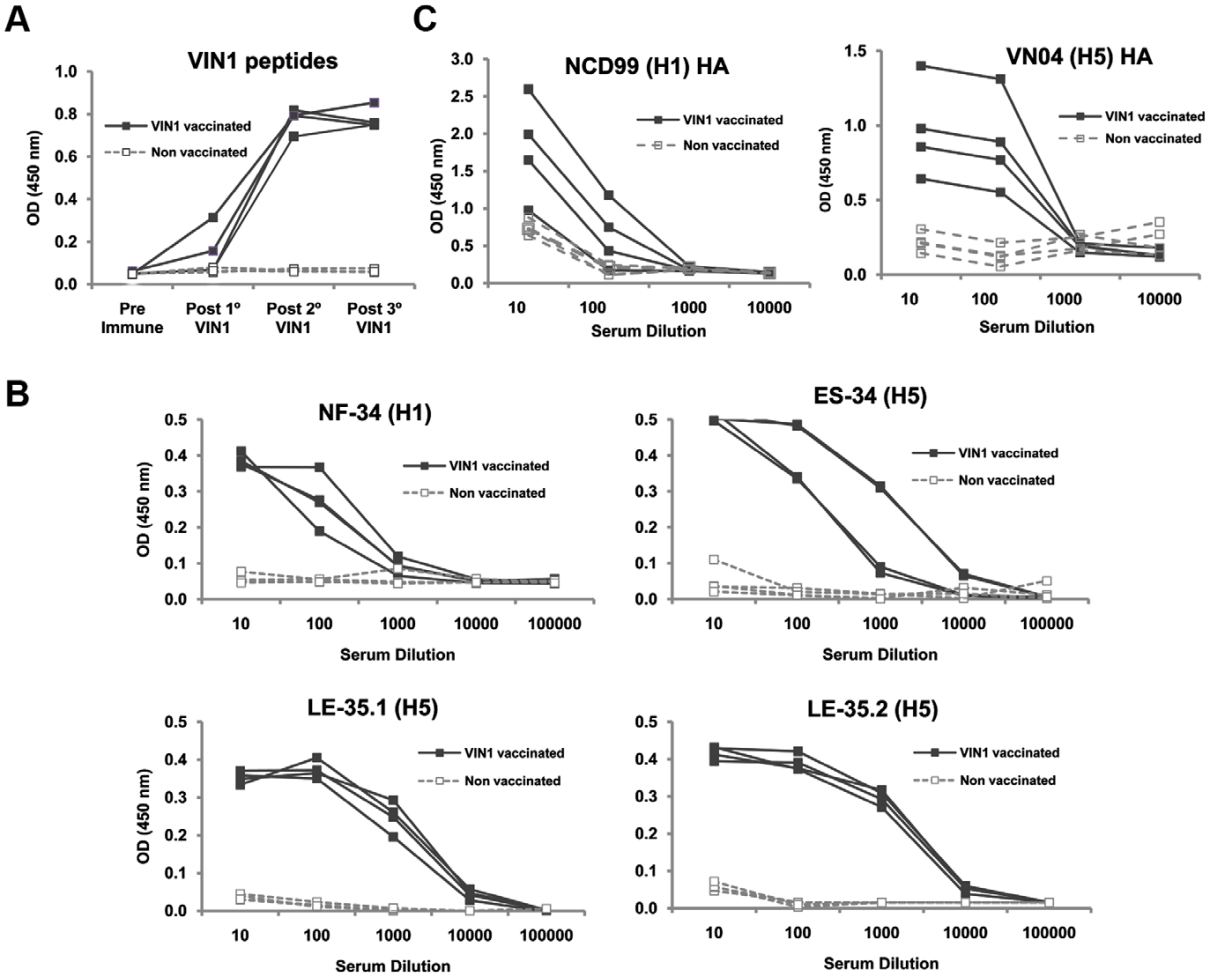
VIN1-peptide cocktail acts as a potent immunogen and the elicited sera reacts with different hemagglutinin subtypes and against VIN1-peptides. (A) Sera from individuals were obtained 15 days after each immunization and were tested for binding to a mixture of the VIN1-peptides (serum dilution 1∶100) by ELISA. (B) Sera from individual pigs were obtained 15 days after the third immunization and were serially diluted and tested for binding to each single peptide by ELISA and (C) Sera described in B) were tested for binding to H5- or H1- recombinant hemagglutinin by ELISA.

The pig is a good model not only to study influenza pathogenesis and therapy [9], but also for developmental immunology [10]. Thus, to confirm the bioinformatic predictions related to the capacity of VIN1-peptides to elicit humoral responses, four of the influenza-seronegative pigs were immunized three times with the VIN1-peptide mixture with two week intervals between immunizations. Four extra-pigs (also negative for IV antibodies) were inoculated with saline solution and remained as negative controls during the assay. To evaluate the ability of the VIN1-peptide cocktail to elicit antibodies, sera obtained 15 days after each immunization were tested against the peptides. Results obtained by ELISA showed that the immunization was efficient since every single immunized pig produced specific antibodies against the VIN1-peptide pool, detectable after the second immunization (Fig. 1A). VIN1-peptides also elicited high antibody titres against each one of the single peptides included in the vaccine (Fig. 1B). In correlation with the high specificity shown in the peptide-ELISA, sera from peptide-immunized pigs specifically reacted against the purified hemagglutinin protein of H5- and H1- subtypes, with only one serum from the V1N1 group showing background OD values in the H1-ELISA (Fig. 1C).

**Figure 2:**
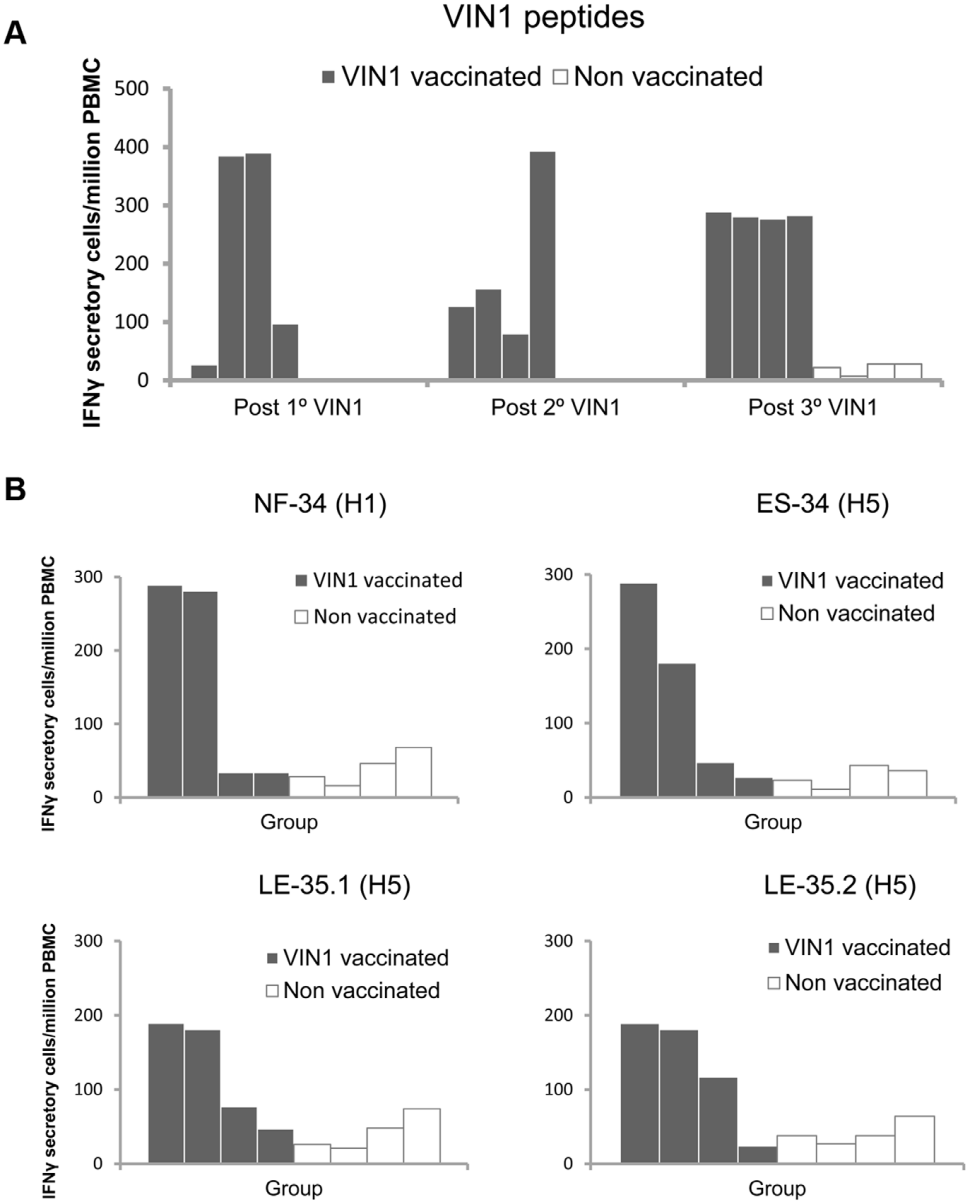
VIN1-HA1 derived peptides immunization induces strong T-cell responses in pigs. (A) Kinetics of the VIN1 peptide-specific T-cell responses induced 15 days after all immunizations measured by IFNγ-ELISPOT. (B) Specific T-cell responses induced 4 weeks after the third immunization were tested for each single peptide by IFNγ-ELISPOT.

Finally, we were intrigued to find that VIN1-peptides also had the ability to induce T-cell responses. PBMC isolated from VIN1-immunized pigs specifically secreted IFN-γ in response to *in vitro* stimulation with VIN1-peptide cocktail (Fig. 2). First, we noted that VIN1-PBMC specifically secreted IFN-γ two weeks after the first immunization. Second, a homogeneous T-cell activity against the V1N1-peptide cocktail was detected between animals after the third immunization (Fig. 2*A*). And third, that all peptides were recognized by the specific T-cell induced (Fig. 2B). These results demonstrated that VIN1-peptides could act as productive immunogens in pigs.

### VIN1 Peptide Immunization Partially Prevent pH1N1 Virus Replication in BAL

As previously reported, the pig can be used to evaluate the protection of experimental pH1N1 influenza vaccines since they are natural receptive hosts for this virus subtype [11]. Aiming to evaluate the protective potential of our vaccine prototype, VIN1 peptide-vaccinated and control pigs were subjected to intranasal challenge with 10^6^ TCID_50_ of pH1N1 IV. The pH1N1 virus differs in three amino acids from the H1-peptide (NF-34) used in the vaccine (Table 1).

**Figure 3:**
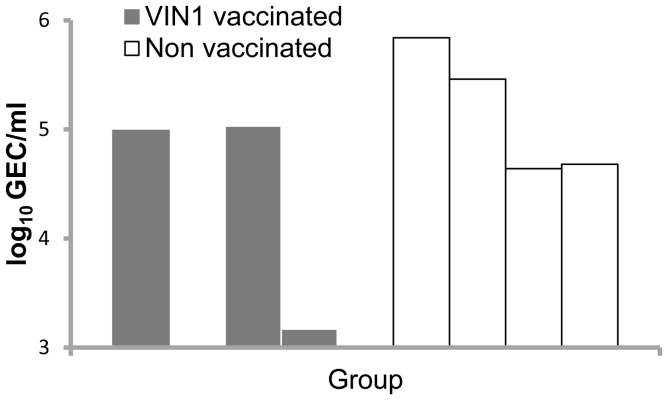
Immunization with VIN1-HA1 partially protects pigs *in vivo* against heterologous challenge with pH1N1. Influenza viral RNA quantification in BAL was performed by RT-qPCR at 6 dpi, corresponding to necropsy day. Bars indicated positive samples in genome equivalent copies (GEC) per ml of BAL. The detection limit in the assay was 3 log_10_ GEC/ml.

Intranasal infection of control pigs caused a subclinical infection and minor hystopathological changes. Moreover, mild to moderate BIP was recorded at necropsy (6 dpi), albeit virus was recovered from BAL at this time-point (Fig. 3). These results are in concordance with previously reported data obtained using colostrums-deprived pigs [11]; therefore, validating the use of seronegative conventional animals for vaccine testing. We did not detect differences in the severity of the lesions in lungs of vaccinated and non vaccinated animals. However, in contrast with control pigs, 2 out of 4 VIN1-peptide vaccinated pigs showed no or less viral RNA in their BAL (Fig. 3), which demonstrates a partially protective effect of our experimental vaccine.

### VIN1 Peptides Induce Antibodies and T-cells that Specifically Recognize the pH1N1 Virus

**Figure 4:**
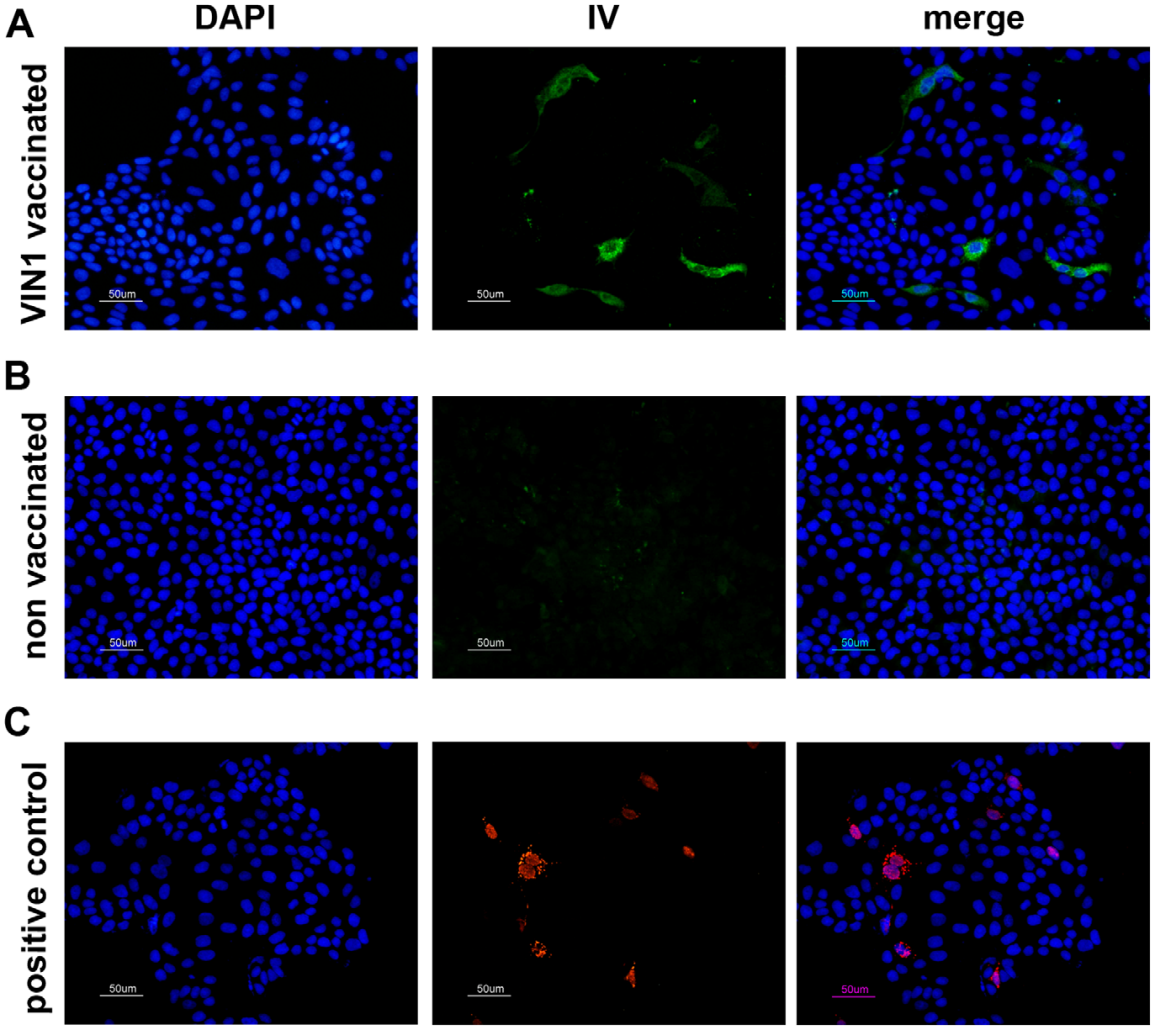
VIN1-sera recognize pH1N1 virus *in vitro.* Indirect immunofluorescence of pH1N1-infected MDCK cells at 16 hpi using as primary antibody: (A) the serum from one pig (representative of the group), immunized three times with VIN1-peptides; (B) the serum from one negative control pig (representative of the group), immunized three times with PBS; and (C) A monoclonal antibody against the NS-1 protein was used as control for the infection (right panel).

**Figure 5:**
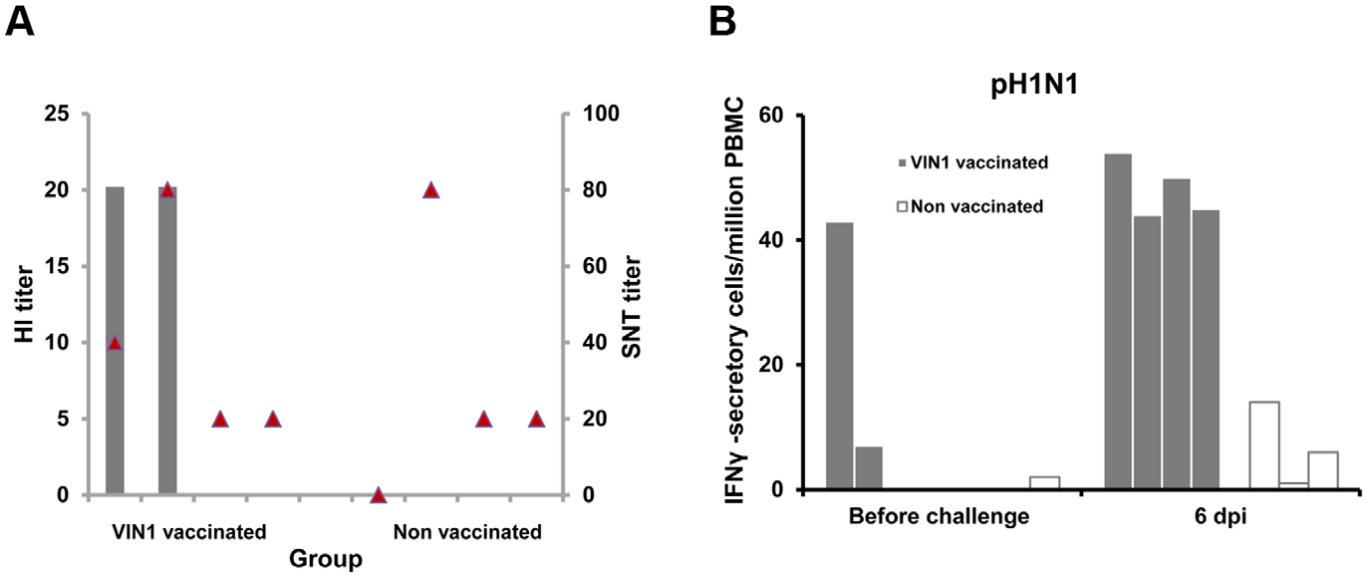
Immunization with the VIN1-HA1 peptide induces specific antibodies and T-cells against the heterologous pH1N1 virus. (A) HI and SNT titers obtained with sera from pigs immunized either with the VIN1-peptides or with saline solution (control), at 6 dpi with the pH1N1 virus. Grey bars represent HI titres and red triangles show SNT. (B) IFNγ-ELISPOT using pH1N1 virus as stimulus and PBMCs from pigs immunized either with the VIN1-peptides or with saline solution (control). The assay was done using PBMCs isolated either before the infection with the pandemic H1N1 virus or at 6 dpi.

In an attempt to correlate the protection provided from the immunological outcome induced by our vaccine, sera from immunized and control pigs were used to evaluate their capability to *in vitro* recognize the pH1N1. Sera obtained before the challenge from pigs vaccinated with VIN1-peptides, specifically detected pH1N1 infected-MDCK cells, as shown by indirect IF (Fig. 4 panel A). As expected, sera from control animals showed no reaction (Fig. 4 panel B); thus, demonstrating the ability of the peptide-induced antibodies to specifically identify the virus. Importantly, every single cell infected by pH1N1 was also recognized by the specific NS1-monoclonal antibody (Fig. 4 panel C) confirming the specificity of the reactions. Furthermore, sera from 2 of the pigs immunized with VIN1-peptides showed detectable HI activity, albeit at low titre and only those obtained at 6 dpi (Fig. 5A). As expected, sera from the control pigs did not show any specific response even at 6 dpi, which confirms the efficacy of our experimental vaccine to prime for viral-specific antibody responses. Regarding the presence of SNT antibodies, no significant differences were observed between the animal groups, at least at day 6pi (Fig. 5A).

As occurred for the antibodies, the induced T-cell responses measured by IFN-γ ELISPOT, not only specifically recognized the synthetic peptides, but also the pH1N1 virus. Thus, before the challenge only one out of four of the VIN1-vaccinated pigs showed detectable T-cell responses in response to *in vitro* stimulation with the inactivated pH1N1, while all vaccinated pigs responded at 6 dpi (Fig. 5B).

### VIN1 Peptides Induce Antibodies that Recognize Distinct Viral Subtypes

**Figure 6:**
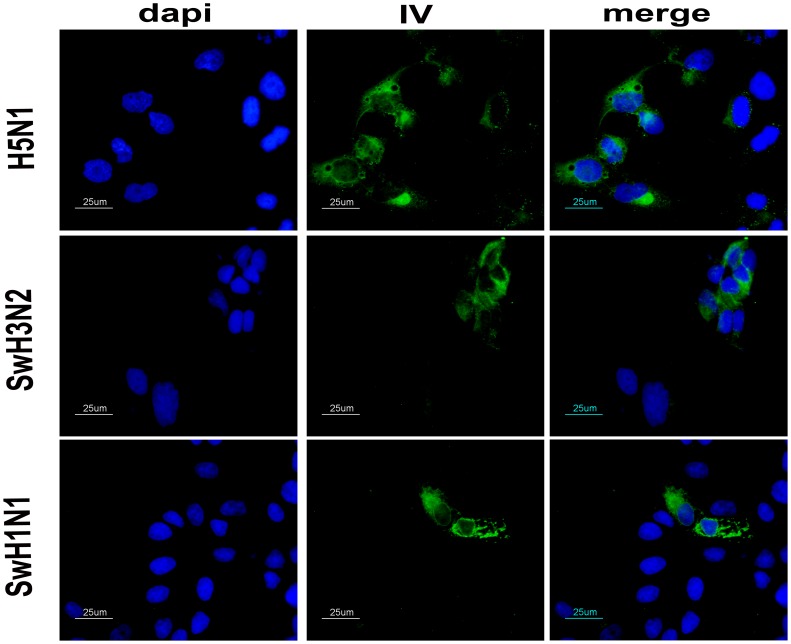
VIN1-sera recognize distinct viral subtypes. Indirect immunofluorescence of either H5N1, SwH3N2 or SwH1N1-infected MDCKs cells at 16 hpi using as primary antibody the serum from one pig (representative of the group), immunized three times with VIN1 peptides.

Current influenza vaccines protect mostly against homologous virus strains. The presented VIN1-peptide cocktail did not confer efficient neutralizing antibodies and only one pig did not show viral RNA in BAL 6 dpi. However, an IF was performed to demonstrate that vaccination elicits antibodies that recognize different viruses. VIN1-sera obtained after three immunizations specifically detected SwH1N1 and H5N1 infected-MDCK cells, as shown by IF (Fig. 6). Furthermore, antibodies elicited after VIN1-immunization specifically detected SwH3N2 (Fig. 6).

To further investigate the antibody response, an HI assay was performed against the same virus subtypes: H5N1 HPAIV, SwH1N1 IV and SwH3N2 IV. No inhibition activity was recorded against any of the mentioned virus for any sera.

## Discussion

The search for universal vaccines against influenza viruses is a must. Most efforts have been focussed on driving the immune response against well conserved epitopes or proteins of IV, such as the **influenza ion channel M2 protein**, and conserved epitopes from the influenza NP and **matrix 1 (M1)** [12], [13], [14]. More recently, the potential use of highly conserved synthetic peptides from HA2 as an efficient vaccine in mice has also been demonstrated [15]. In this report, we show evidence of the potential use of conserved HA1 peptides in future vaccine formulations using conventional pigs.

Peptides derived from the HA1-VIN1 domain were selected by ISM [6], [7] and were used for the immunization carried out in the present study. As predicted, immunization of pigs with VIN1-peptides induced specific anti-VIN1 peptides antibodies that recognized the VIN1-peptides (Fig. 1A and 1B), the H1 and H5 recombinant proteins (Fig. 1C) and also the heterologous pH1N1 IV (Fig. 4). Even though it was not predicted, VIN1-peptide immunization was also able to induce T-cell responses in every single conventional pig that, again, not only recognized the specific peptides but also the heterologous pH1N1 IV. Interestingly enough, not all peptides seemed to be equally recognized, with both the NF-34 and ES-34 from the H1 and H5 hemagglutinin, respectively, being optimally recognized. The fact that these two epitopes are located in equivalent regions within the primary structure of the HA1 subunit, validate even more the ISM predictions.

An **ideal vaccine should elicit** both humoral and cellular responses in the context of highly variable Major Histocompatibility Complex (MHC), which is what we found with our vaccine. The fact that swine and human MHC complexes are remarkably similar [16], opens avenues for the extrapolation of these and future results for human medicine.

We observed an increase in virus clearance after the challenge with pH1N1 virus, which differs in 3 amino acids from NF-34 (the H1-peptide used in the VIN1-vaccine) (Table 1), in 2 out of 4 of the immunized pigs. This also opens new expectations for the use of VIN1-modified peptides in future vaccine formulations. Apart from sequence diversity, there is a clear lack of correlation between the protection observed and the immune responses detected at the individual level. Intriguingly, as soon as at day six post infection, only 2 pigs (pigs 1 and 2 from the V1N1 peptide-immunized group) showed concomitant detection of neutralizing and HI activity that did not totally correlate with protection. While pig 2 showed a clear reduction in viral load, pig 1 showed virus titres indistinguishable from those found in the control group. Although disappointing, our results seem to point towards the very important role of T-cells in the protection afforded which could be an important tool for developing more efficient vaccines for the future. Thus, the partial protection observed might correspond with the induction of non-detectable specific cytotoxic T-cell activity (CTL), as has been reported before for influenza [17], [18] or with any other kind of T-cell activity independent from the induction of IFN-γ that might be involved in cross-protection [19]. We are currently addressing these issues, including the identification of shorter specific CTL-peptides.

The length of the peptides used, as well as the fact that the T-cells specifically secreted IFN-γ in response to *in vitro* stimulation with both the NF-34 peptide and the pH1N1 IV, point towards the induction of specific CD4+-T cells in every single vaccinated farm pig and independent of its SLA II haplotype. This, together with the fact that the specific antibodies induced are also able to recognize the pH1N1 virus, seem to validate the use ISM to optimize the prediction of highly conserved epitopes with better protective ability and to design future vaccine formulations, capable of inducing concomitantly, universal B and T-cell responses against H1N1 influenza viruses [20].

Unexpectedly, the reduction in the viral loads shown by pigs 2 and 4 did not correlate with less severity in the lung lesions. All pigs from either control or immunized groups show indistinguishable minor hystopathological changes. Despite the fact that these results could reflect a limitation of our T cell-centric vaccines to reduce disease, pigs might not be ideal models to test so, mainly because of the mild disease found after pH1N1 infection. For that reason, our hypothesis are also being tested in mice and chickens, which are ideal models for the characterization of the protective capability of experimental vaccines against an infection with highly pathogenic H5N1 IV; most probably, the responsible of future pandemic episodes [21].

## Materials and Methods

### Ethics Statement

All experiments with the pH1N1 IV were performed at the Biosafety Level 3 facilities of the *Centre de Recerca en Sanitat Animal* (CReSA-Barcelona). Sample from the patient infected by pH1N1 IV was coded prior to isolating the virus to ensure anonymity. For this reason, the Ethical and Animal Welfare Committee of the *Universitat Autònoma de Barcelona* (UAB) exempted this study from the requirement to have the consent of the patient, who was infected with pH1N1 IV. The present study was performed in accordance with the Guidelines of the Good Experimental Practices and under the supervision and approvement of the Ethical and Animal Welfare Committee of the UAB (*Permit Number:* DMAH-5796).

### Animal Experimental Design

A total of eight 8-wk-old conventional crossbreed pigs from a three-way cross (Duroc x Landrace hybrids paired with Pietrain boars) seronegative against influenza A virus were immunized three times two weeks apart. We immunized the pigs with either 15 µg of the VIN1-peptide cocktail (3.5–4 µg of each peptide) or saline solution in complete Freund’s adjuvant (first immunization), incomplete Freund’s adjuvant (second dose) and without adjuvant (last dose), by i.m. administration. Four weeks after the second boost, the pigs were intranasally inoculated with 10^6^ TCID_50_ of the pH1N1 virus. Animals were monitored daily for flu-like clinical signs. Sera and peripheral blood mononuclear cells (PBMC) obtained before each immunization, before the challenge and at 6 days post-infection (dpi), were used to detect specific humoral and cellular responses, respectively. Animals were euthanized at 6 dpi and a complete necropsy was carried out for each animal. Bronchoalveolar lavages (BAL) from the right lung of each pig were performed in 200 ml of PBS 1× immediately after post-mortem examination. BAL were frozen at −80°C until their use for viral RNA extraction and quantification. For histopathological analysis, samples from lung (apical, middle and diaphragmatic lobes), nasal turbinate and trachea were collected and fixed by immersion in 10% neutral buffered formalin. In the lung, broncho-interstitial pneumonia (BIP) intensity was assessed by means a semi-quantitative scoring (0 to 3, indicating lack of, mild, moderate or severe pneumonia lesions, respectively), as previously described [11].

### Virus and Purified Hemagglutinins

Viruses used were pH1N1 virus (the pandemic swine-origin A/Catalonia/63/2009 H1N1 IV) [GenBank GQ464405-GQ464411 and GQ168897], SwH1N1 virus (A/Swine/Spain/003/2010 H1N1 IV) [GenBank JQ319725 and JQ319727], SwH3N2 virus (A/Swine/Spain/001/2010 H3N2 IV) [GenBank JQ319724 and JQ319726] and H5N1 HPAI virus (A/great crested grebe/Basque Country/06.03249/2006 H5N1 HPAIV) [GenBank EU636810 and EU636811]. After propagation at 37°C in the allantoic fluid of 11-day-old embryonated chicken eggs from a specific-pathogen-free flock, the infectious virus titre was determined in Madin-Darby Canine Kidney (MDCK, ATCC CCL-34) cells and measured as tissue culture infectious doses 50% (TCID_50_) by following the Reed and Muench method [22]. Purified hemagglutinin for A/VietNam/1203/04 (H5) and A/New Caledonia/20/99 (H1) were purchased from Abcam.

### Peptide Synthesis

Four peptides were designed based on ISM predictions [6], [7] and were mixed and used to immunize conventional pigs. The selected peptides were highly conserved and mapped to the flanking region of the HA1 within the VIN1 domain. Two peptides (LE-35.1 and LE-35.2) were derived from A/Hong Kong/213/03 (H5N1) [GenBank AB212056] and one (ES-34) from A/Egypt/0636-NAMRU3/2007 (H5N1) [GenBank EF382359]. The fourth peptide (NF-34) was derived from the HA1 of the human A/South Carolina/1/18 (H1N1) strain [GenBank AF117241]. The peptides were produced by GL Biochem (Shanghai) Ltd. Sequences from the synthetic peptides (thereafter referred as VIN1-peptides) are shown in Table 1.

### Quantitative Real Time RT-PCR (RT-qPCR)

Viral RNA quantification using TaqMan RT-qPCR was performed in BAL. Viral RNA was extracted with QIAamp Viral Mini kit (Qiagen, Inc.). Amplification of a matrix (*M*) gene fragment was carried out using primers, probe, one-Step RT-PCR Master Mix Reagents (Applied Biosystems) and amplification conditions as described previously by Busquets *et al.* 2010 [11] in Fast7500 equipment (Applied Biosystems).

### Influenza Nucleoprotein (NP)-specific ELISA

Sera from animals before starting the experiment were examined for the presence of specific antibodies against influenza NP using the ID Screen® Influenza A Antibody Competition ELISA (ID VET, France), following manufacturer’s instructions. Pig serum samples were used at 1∶100 dilution. Known positive and negative sera were used as test controls.

### Peptide-specific ELISA

A peptide-based ELISA method was developed for the evaluation of the presence of specific antibodies in serum samples. Briefly, 96 well plates (Costar, Corning Incorporated) were coated with 1 µg/ml of each peptide individually, the VIN1-peptides cocktail or H5−/H1- purified hemagglutinin in coating buffer (sodium bicarbonate 0.1 M) overnight at 4°C. After blocking with 1% casein/PBS 1× for 1-h at 37°C, serum from individuals were added to the coated plate diluted at 1∶100 or titrated with 10-fold dilutions (starting from 1∶10), followed by 2-h incubation at 37°C. Plates were washed four times with PBS 1×/0.1% Tween20 and anti-pig IgG (whole molecule)-Peroxidase (Sigma) diluted 1∶20,000 was added to wells followed by 45 min incubation at 37°C. After washing the plates four times (PBS 1×/0.1% Tween20), fifty µl of 3,3′,5,5′-tetramethylbenzidine (TMB) substrate solution were added to the wells and allowed to develop for 8–10 min at room temperature (RT) protected from light. Optical density (OD) was measured at 450 nm.

### Haemagglutination Inhibition (HI) Assay

An HI assay was performed following the standard procedures [23] using chicken red blood cells (RBC) and 4 haemagglutination units of either pH1N1 IV, SwH1N1 IV, SwH3N2 IV or H5N1 HPAIV. To avoid unspecific inhibitions, sera from individuals were treated prior to use. Briefly, one volume of serum samples was treated overnight at 37°C with four volumes of Receptor Destroying Enzyme (Sigma) solution (100 U/ml). Next day, serum samples were incubated for 30 min at 56°C after the addition of five volumes 1.5% sodium citrate. Finally, one volume of a 50% suspension of RBC was added and incubated for 1-h at 4°C. Known positive and negative sera were used as controls. HI titres of ≥20 were considered positive.

### Seroneutralization (SNT) Assay

A SNT assay was done following the protocol described by Sirskyi and collaborators (2010) [24], with some modifications. Serum samples were diluted serially and incubated with 100 TCID_50_ of pH1N1 virus for 2-h at 37°C. The mixture was then added to 10^5^ MDCK cells/well and incubated overnight. After two washes with PBS 1×, the cells were fixed with cold 80% acetone for 10 min. Cells were air-dried, washed five times with PBS 1×/0.05% Tween-20 and incubated at RT for 1-h and a half with biotinylated influenza A anti-NP primary antibody (CAT # MAB8252B, Millipore, CA) diluted 1/2,000 in 5%FBS/PBS 1×. Plates were then washed five times with PBS 1×/0.05% Tween-20 and incubated 30 min in the dark with HRP-conjugate streptavidin (Millipore, CA) diluted 1/10,000 in 5%FBS/PBS 1×. Finally, after five washes with PBS 1×/0.05% Tween-20, TMB substrate (Sigma) was added to develop the reaction and stopped with Stop-solution H_2_SO_4_ (1N). Plates were then read at 450 nm.

### IFN-γ ELISPOT Assay

An IFN-γ ELISPOT was performed as previously described [25], with some modifications. Briefly, PBMC were isolated from whole blood by Histopaque-1077 gradient (Sigma). Ninety-six-well plates (Costar, Corning Incorporated) were coated overnight with IFNγ-capture antibody (P2G10 clon, BD Pharmingen) diluted 1∶100. After blocking the plates 1-h at 37°C, 500,000 PBMC/well were seeded and stimulated with either 2 µg/ml of VIN1-peptide cocktail or individually, or with 10^5^ TCID_50_ of inactivated pH1N1 IV per well for 20-h. Cells were removed and a biotin mouse anti-pig IFN-γ detection antibody (BD Pharmingen), diluted 1∶1,000, was used followed by streptavidin-peroxidase (0.5 µg/ml). Insoluble TMB blue (Calbiochem) was added as final substrate. Positive spots were counted using a microscope.

### Immunofluorescence Microscopy

MDCK cells (300,000 cells/well) were either mock infected or infected with pH1N1 IV, SwH1N1 IV, SwH3N2 IV or H5N1 HPAIV for 16-h at a MOI of 0.01. Cells were fixed with 4% paraformaldehyde and permeabilized with 0.5% Triton X-100. The cells were then blocked with 3%BSA/PBS 1× for 1-h and incubated with the sera from the pigs (1∶100) for 1-h in the blocking solution at RT. After three washes with PBS 1×, the cells were incubated with anti-IgG pig:FITC antibody (1∶300) (Jackson Immunoresearch Europe LTd) for 1-h in blocking solution at RT. Finally, nuclei were counterstained with DAPI (1 µg/ml) and coverslips were mounted with Vectaschield. Protocol was modified from the previously described by Ballester *et al.* 2011 [26]. Fluorescence images were viewed on a Nikon eclipse 90i epifluorescence microscope equipped with a DXM 1200F camera (Nikon Corporation, Japan). The images were processed by using the Image J v1.45l software (http://rsb.info.nih.gov/ij).
